# The effect of thickness and elastic modulus of the anterior talofibular ligament on anterior ankle joint stiffness: A subject-specific finite element study

**DOI:** 10.3389/fbioe.2023.1175347

**Published:** 2023-04-25

**Authors:** Linjing Peng, Lu Yu, Jingyi Jia, Yaokai Gan, Angang Ding, Ping Xiong, Yichen Zhao, Yifei Yao

**Affiliations:** ^1^ School of Biomedical Engineering, Shanghai Jiao Tong University, Shanghai, China; ^2^ Engineering Research Center of Digital Medicine of the Ministry of Education, Shanghai Jiao Tong University, Shanghai, China; ^3^ Department of Orthopedics, Shanghai Ninth People’s Hospital, School of Medicine, Shanghai Jiao Tong University, Shanghai, China; ^4^ Department of Ultrasound, Shanghai Ninth People’s Hospital, School of Medicine, Shanghai Jiao Tong University, Shanghai, China

**Keywords:** anterior talofibular ligament, anterior ankle joint stiffness, ultrasound elastography, finite element analysis, lateral ankle sprains

## Abstract

Ankle sprain is a frequent type of sports injury leading to lateral ligament injury. The anterior talofibular ligament (ATFL) is a primary ligamentous stabilizer of the ankle joint and typically the most vulnerable ligament injured in a lateral ankle sprain (LAS). This study aimed to quantitively investigate the effect of the thickness and elastic modulus of ATFL on anterior ankle joint stiffness (AAJS) by developing nine subject-specific finite element (FE) models under acute injury, chronic injury, and control conditions of ATFL. A 120 N forward force was applied at the posterior calcaneus leading to an anterior translation of the calcaneus and talus to simulate the anterior drawer test (ADT). In the results, the ratio of the forward force to the talar displacement was used to assess the AAJS, which increased by 5.85% in the acute group and decreased by 19.78% in the chronic group, compared to those of the control group. An empirical equation described the relationship between AAJS, thickness, and elastic modulus (R-square 0.98). The equation proposed in this study provided an approach to quantify AAJS and revealed the effect of the thickness and the elastic modulus of ATFL on ankle stability, which may shed light on the potential diagnosis of lateral ligament injury.

## Introduction

Ankle sprain is one the most common type of sports injuries from the epidemiological data (Swanson et al., 2022), approximately 85% of which are LAS (Ferran and Maffulli, 2006). The injuries of the lateral ankle joint are typically due to sudden excessive inversion, which is combined with a pronounced plantarflexion and internal rotation of the ankle joint complex (Fong et al., 2012). The initial LAS can weaken ankle stability leading to approximately 40% recurrent ankle sprains in the long term (Miklovic et al., 2018), known as chronic ankle instability (CAI) (Herzog et al., 2019). It can compromise the patient’s ability to participate in physical activities and ultimately lead to abnormal joint function, which can develop into osteoarthritis (Golditz et al., 2014).

Lateral ankle ligaments include the anterior talofibular ligament (ATFL), the calcaneofibular ligament (CFL), and the posterior talofibular ligament (PTFL). ATFL was the most mechanically vulnerable ligament (Kumai et al., 2002), injury of which was involved in 85%–100% of LAS (Ferran and Maffulli, 2006; Swanson et al., 2022), while CFL was involved in 35% and PTFL was involved in 10%. Extending from the anterior border of the lateral malleolus of the fibula to the neck of the talus, ATFL was also the structurally weakest in the lateral ankle ligaments (Khawaji and Soames, 2015). At heel strike, the forced ankle inversion-supination destabilizing the ankle joint was considered a possible injury factor of ATFL tear (Walls et al., 2016). Studies have reported various pathological features of ATFL integrity assessed through MRI and arthroscopic surgery, with some cases showing as thin or absent, while others exhibiting thickening (Kanamoto et al., 2014; Jung et al., 2017). The shear wave velocity (SWV) in acoustic radiation force impulse (ARFI) imaging, which represents the stiffness of the materials, showed that injured ATFL in the acute and subacute groups were significantly softer than those of non-affected ATFL, while the ATFL in the chronic group were slightly stiffer than that of the non-affected ATFL (Chen et al., 2021).

For LAS patients, ADT, a physical test, was performed to assess the integrity and laxity of the lateral collateral ligaments in the sagittal plane. The evaluation of the ATFL rupture through the rotational ankle laxity (i.e., pivot test) has been proposed (Guerra-Pinto et al., 2018). This approach relied on the uncompromised medial ligaments that block any pure anterior translation of the talus underneath the tibia (Bulucu C et al., 1991). However, the low inter-observer variability of ADT and patient variability limited the applications in diagnosing ATFL injury (Murahashi et al., 2023).

Previous studies investigated lateral ankle ligament injury in LAS in different ankle postures. Approximately 10 degrees of rotational mal-alignment in the subtalar inversion during the swing phase may result in lateral ankle ligament complex lesions, as investigated in a cadaveric study of simulated gait in a material testing machine (Konradsen and Voigt, 2002). A numerical study of the ankle joint indicated that an internal rotation position of 15 degrees was identified as the position which led to the most vulnerable ATFL (Shin et al., 2012). However, it should be noticed that the defect of lateral ankle ligaments can also affect ankle stability in the long run. Previously, the three lateral ankle ligaments were separately sectioned to assess the mechanical contribution of the individual ligament with regard to ankle joint stability in a cadaveric experiment (Li et al., 2019). In-silico simulation using the FE model could be a powerful tool to analyze the mechanical behavior of the lateral ankle ligaments in ankle joint stability. The non-linear FE model of a normal foot structure could determine the stress and strain of the lateral ankle ligaments during ADT (Pekedis et al., 2011). Furthermore, previous studies provided insight into the biomechanical mechanism of lateral ankle ligament injury and ligament mechanical behavior on joint stability but paid little attention to the effects of the material properties and morphologies of injured ATFL alone on ankle stability. Biomechanics of ATFL injury in ankle instability without complete ligament rupture should also be considered, as microtears or slight tears are more likely to occur compared to complete ruptures (Cavazos and Harkless, 2021).

Thus, this study aimed to evaluate the influence of the thickness and the elastic modulus changes in different ATFL injury conditions on AAJS via three-dimensional FE analysis. We hypothesized that the AAJS decreased with the decrease of the thickness and the elastic modulus of ATFL.

## Methods

### Participants

Nine participants were recruited for this study. Six LAS patients were recruited in the orthopedics department at Shanghai Ninth People’s Hospital, Shanghai Jiao Tong University School of Medicine, from March 2021 to October 2022. The injuries were recognized as acute if the injury duration was less than 3 months; otherwise, they were recognized as chronic (Hubbard and Hicks-Little, 2008). There were 3 patients (1 man; 2women) in the acute group and 3 patients (2 men; 1woman) in the chronic group. Three participants in the control group (1 male; 2 female) were recruited from the community without any lower limb disease history. All the participants were older than 18 years old and submitted informed consent for this study. This study was approved by the Shanghai Ninth People’s Hospital, Shanghai Jiao Tong University School of Medicine Ethics Committee (SH9H-2021-TK432-1) in China.

### Model reconstruction

The subject-specific ankle joint three-dimensional geometric model consisting of the distal end of the tibia and fibula, as well as the talus and calcaneus, was reconstructed using MR image sequences. Three-dimensional structures of bones and ATFL were segmented in MIMICS (Materialise, Leuven, Belgium) with forty axial slices (2.0 mm slice interval, 480 pixels width, 480 pixels height, and 0.33 mm pixel size). The surface models in MIMICS were then turned into solid models in SolidWorks (Dassault Systemes, Waltham, MA, USA).

**FIGURE 1:**
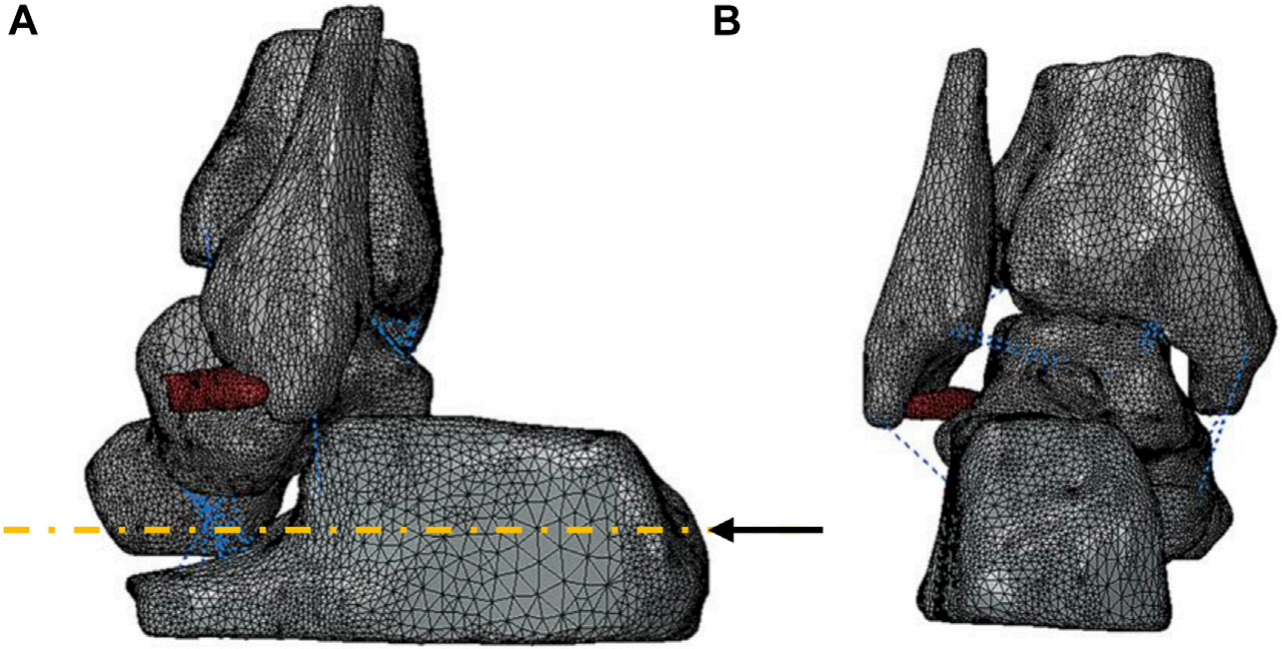
Three-dimensional geometry model of the ankle joint: **(A)** lateral view and **(B)** posterior view of the bones and ATFL represented in grey and red, respectively. A black arrow represented the vector of the forward force. The blue dotted lines showed the eight ligaments established in 1D truss elements.

The ankle FE models were established in Abaqus 2020 (Dassault Systemes, Waltham, MA, USA), including the three-dimensional structures of the tibia, talus, fibula, calcaneus, and ATFL and 1D truss of the eight ankle ligaments. Eight ligaments, including CFL, PTFL, posterior inferior tibiofibular ligament (PTiFL), posterior tibiotalar ligament (PTTL), tibial collateral ligament (TCL), anterior tibiotalar ligament (ATTL), anterior tibiofibular ligament (ATiFL), and interosseous talocalcaneal ligament (ITCL), were only able to sustain tension. Bones and ATFL meshed as the C3D10 elements. The material property of the bones was assumed as homogeneous and isotropic (Table 1) (Gefen et al., 2000). The elastic response function $T\left(\varepsilon \right)$ of eight ligaments was described as,

$$T\left(\varepsilon \right)=\frac{A\left(e^{B\varepsilon }-1\right)}{n}$$ (1)

where n was the number of wires; A and B were the coefficients (Funk et al., 2000), as shown in Table 2. Meanwhile, ITCL, PTFL, and PTTL were modeled as 10, 2, and 4 wires with the truss property, respectively. The remaining ligaments were modeled as single wires with the truss property (Figure 1).

**TABLE 1:** Material properties of bone were assumed from the reference; ATFL shear wave velocities were measured using ARFI; Elastic modulus values applied in the FE models were estimated according to the shear wave velocities; Poisson’s ratio were assumed according to the reference.

Material	Shear wave velocity/m•s^-1^	Elastic modulus (FEA)/MPa	Poisson’s ratio
Bones	—	7,300	0.3
ATFL (control 1)	3.09	260.5	0.49
ATFL (control 2)	3.11	263	0.49
ATFL (control 3)	2.97	241	0.49
ATFL (acute 1)	2.77	209.8	0.49
ATFL (acute 2)	2.93	233.6	0.49
ATFL (acute 3)	2.83	218.1	0.49
ATFL (chronic 1)	3.31	298.3	0.49
ATFL (chronic 2)	3.03	249.9	0.49
ATFL (chronic 3)	3.29	295.1	0.49

ATFL, anterior talofibular ligament.

**TABLE 2:** Material coefficient of eight ligaments.

Ligament	Coefficient A	Coefficient B
ATiFL	5.52	22.63
ATTL	2.06	20.11
CFL	0.2	49.63
PTFL	0.14	44.35
PTiFL	6.87	20.07
TCL	0.51	45.99
PTTL	1.34	28.65
ITCL	1.34	28.65

ATiFL, anterior tibiofibular ligament; ATTL, anterior tibiotalar ligament; CFL, calcaneofibular ligament; PTFL, posterior talofibular ligament; PTiFL, posterior tibiofibular ligament; TCL, tibio-calcaneal ligament; PTTL, posterior tibiotalar ligament; ITCL, interosseous ligament.

### Morphological and material properties

All the participants underwent ARFI imaging, a kind of ultrasound elastography, for the measurement of the thickness and the elastic modulus of ATFL. It was performed by an ultrasonographer with expertise in the musculoskeletal system using a Siemens ACUSON S2000 (Medical Solutions USA Inc., Mountain View, CA, USA) with a 9L4 linear array transducer, a merging ultrasound technique being able to quantify the SWV of ATFL *in vivo* non-invasively. During the ARFI imaging, participants were required to sit on the examination table in a seated position and flex their knees to 90 degrees. The ultrasound probe was placed along the longitudinal orientation of ATFL, and the SWV mapping within the region of interest covering ATFL was analyzed by Virtual Touch IQTM software. Considering the irregular geometry of the ATFL, the boundary of ATFL was manually selected on the B-mode image overlapped with the color-mapped SWV image using a custom-made Matlab program. The rainbow color bar in the color-mapped SWV image indicated the RGB values corresponded to SWV values varying from 0.5 m/s to 6.5 m/s. The SWV values within manually selected ATFL regions in all participants were calculated based on the relationship between the SWV value and RGB value, as listed in the first column of Table 1. The Poisson’s ratio of ATFL was assumed as 0.49 indicating incompressible properties of ligaments (Siegler et al., 1988). The elastic modulus of ATFL applied in the nine subject-specific FE models was assumed with the magnification coefficient (Table 1), which was the ratio of the ATFL elastic modulus (255 MPa) reported in the reference (Siegler et al., 1988) to the square of the average SWV value in the control group. The thickness values of the three-dimensional ATFL structure segmented from the MRI images were averaged in five randomly selected positions and compared to the thickness results of the B-mode ultrasound measurements in the same way. The comparison results showed high similarity, with less than a 5% difference.

### Boundary and loading condition

The ATFL was assumed to be attached to the talus and fibula, and the interactions between all bones were modeled through a tangential contact formulation with a friction coefficient of 0.1 (Merkher et al., 2006). The hard contacts constraint in the vertical direction was the non-linear penalty function. The top surfaces of the fibula and talus were fixed in six degrees of freedom.

The ADT simulation loading was undertaken in 2 steps to improve the calculation convergence. Firstly, a 3 mm anterior translation was applied to the calcaneus, aligned with the medial axis of the calcaneus (Figure 1A). Then, a 120 N forward force (Chen et al., 2022a; Tsutsumi et al., 2022) with the same translation vector was applied on the 22 mm^2^ surface area of the posterior calcaneus, leading to drive the anterior translation of the talus and the calcaneus. The ratio of a forward force to the anterior relative displacement of the talar dome with the reference of the tibia was used to assess the AAJS.

### Mesh sensitivity analysis

A mesh sensitivity analysis was performed to define the optimal FE mesh size of three-dimensional parts, while the global element size varied between 1 mm and 5 mm. Anterior talar translation value converged after the element size decreased from 3 to 2 mm with 369150 elements, and the global mesh size was determined as 2 mm in the remaining simulations (Table 3).

**TABLE 3:** The results of mesh sensitivity analysis.

Global mesh size (mm)	Number of mesh	Displacement of the talus (mm)
5	300822	2.24
4	333369	2.19
3	354292	2.19
2	369150	2.17
1	390258	2.16

### Validation of the ankle FE model

**FIGURE 2:**
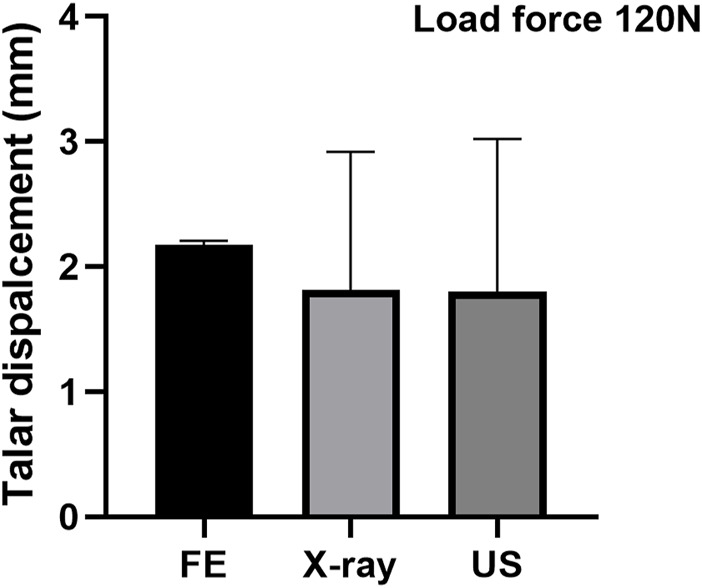
Validation of the FE model by comparing with the reference data measured using ultrasound and X-ray (Tsutsumi et al., 2022).

To validate the simulation results numerically, the talar displacement in the control group under the 120 N forward force applied on the calcaneus was predicted by FE and compared with the reference data measured using ultrasound and X-ray during ADT with a Telos device (Tsutsumi et al., 2022). The mean value of talar displacement (n = 3) was 2.18 mm, which was slightly higher than the reference data (about 1.90 mm) with less than a 15% difference (Figure 2).

### Data and statistical analysis

In this study, the thickness and the elastic modulus of the ATFLs were quantified through ultrasound measurement. Additionally, the AAJS was predicted under the acute injury, chronic injury, and normal conditions of the ATFL in the simulation part.

Statistical analysis was performed using SPSS version 21.0 (IBM Corp. Released 2012) software. All data in this study were presented as mean ± standard deviation (SD). Before the statistical analysis, a Shapiro-Wilk test was performed and confirmed the normality of the variables. The statistical analysis of the thickness of ATFL as well as the elastic modulus were performed using the one-way ANOVA with Tukey’s post-hoc test for pairwise comparison. A *p-value* < 0.05 was considered statistically significant.

## Results

### Demographic data of participants

A total of nine subject-specific FE ankle models were established under acute injury, chronic injury, and intact conditions of the ATFL in this study. The demographic data of all participants were shown in Table 4.

**TABLE 4:** Demographics data of the participants.

	Control	Acute	Chronic
Age (years)	24.33 ± 2.31	28.33 ± 8.51	26.33 ± 5.13
Height (cm)	169.67 ± 11.72	175.33 ± 6.66	170 ± 8.66
Weight (kg)	61.33 ± 9.29	69.67 ± 8.96	67.33 ± 11.24

### Results of morphological and material properties measurement

**FIGURE 3:**
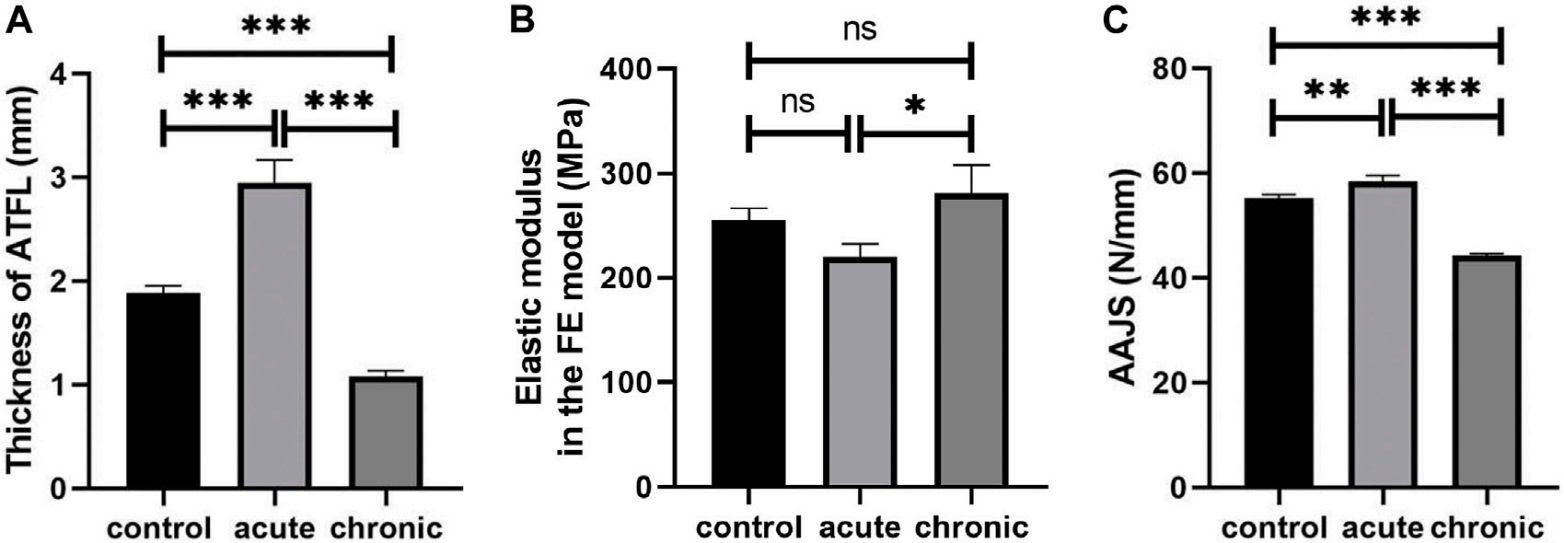
Thickness **(A)** and elastic modulus of ATFL **(B)**, and simulated AAJS **(C)** in different conditions (***p < 0.001, **p < 0.01, *p < 0.05, ns means no significant difference).

The thickness of ATFL was 1.88 ± 0.07 mm in the control group, 2.95 ± 0.22 mm in the acute group, and 1.08 ± 0.06 mm in the chronic group, respectively (Table 5). The thickness of ATFL was significantly increased by 56.4% in the acute group (*p < 0.001*) and decreased by 42.7% in the chronic group (*p < 0.001*), compared to that in the control group, as shown in Figure 3A. The elastic modulus of ATFL in the FE model was 255.0 ± 12.16 MPa in the control group, 220.48 ± 12.10 MPa in the acute group, and 281.7 ± 27.06 MPa in the chronic group, respectively. The differences in the elastic modulus of ATFL with acute and chronic injury conditions were 13.54% and 10.22%, compared to the control group (Figure 3B).

**TABLE 5:** Thickness of ATFL and AAJS of the participants.

	ATFL thickness (mm)	AAJS (N/mm)
Control 1	1.91	55.31
Control 2	1.81	54.30
Control 3	1.94	55.81
Mean _control_	1.88 ± 0.07	55.14 ± 0.77
Acute 1	3.09	59.70
Acute 2	2.69	57.97
Acute 3	3.06	57.42
Mean _acute_	2.95 ± 0.22	58.36 ± 1.19
Chronic 1	1.14	44.61
Chronic 2	1.04	43.80
Chronic 3	1.05	44.28
Mean _chronic_	1.08 ± 0.06	44.23 ± 0.41

### Results of model prediction

The AAJS was 55.14 ± 0.77 N/mm in the control group, 58.36 ± 1.19 N/mm in the acute group (*p < 0.01*), and 44.23 ± 0.41 N/mm in the chronic group (*p < 0.001*), respectively (Table 5). The calculated AAJS was increased by 5.85% in the acute group and decreased by 19.78% in the chronic group, compared to the control group (Figure 3C).

There was a significant positive correlation between AAJS and ATFL thickness (r = 0.919; *p* < 0.001) and a negative correlation between AAJS and elastic modulus (r = −0.794; *p* < 0.01). And we used the following sigmoidal equation to describe the dependence of AAJS on ATFL thickness (T) and elastic modulus (E):

$$AAJS=\frac{k_{1}}{a+e^{bT}}+\frac{k_{2}}{c+e^{dE}}$$ (2)

**FIGURE 4:**
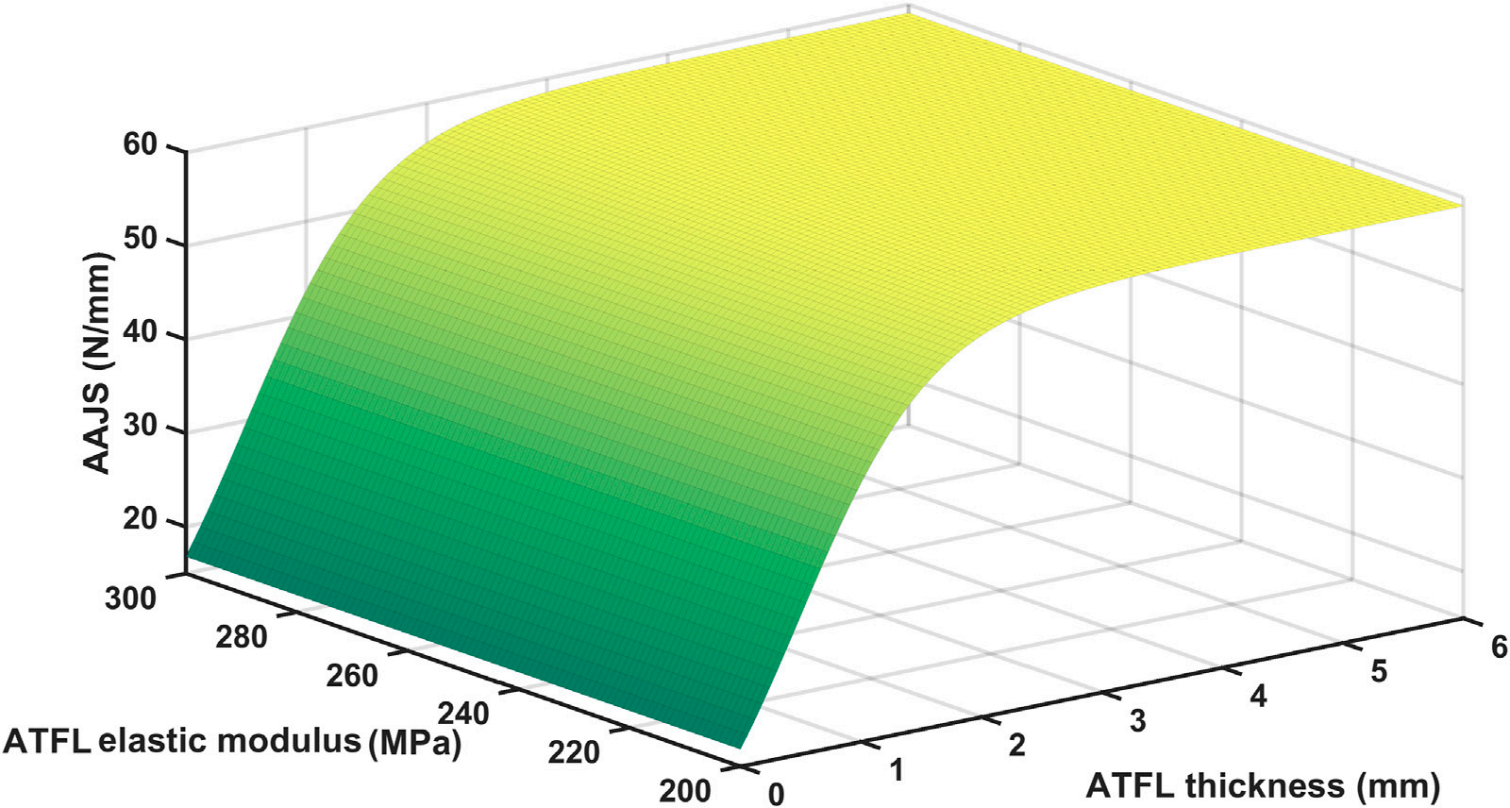
AAJS with different ATFL elastic modulus and thickness.

Least square curve-fitting on the curve in Figure 4 with the above-mentioned equation showed that $k_{1}$ , $k_{2}$ , a , b , c and d equaled to 23.53 N, 0.09754 N mm^-1^·MPa^-1^,0.3983 N, −1.873, 0.3379 MPa, and 0.9134, respectively (R-square 0.98).

## Discussion

The understanding of the ATFL’s role in ankle stability is of vital necessity for effective diagnosis. This study evaluated the effect of the thickness and elasticity of the ATFL on AAJS. Although parametric modeling might be an optional approach (Chen et al., 2022b), the approach may not accurately reflect the physiological and pathological conditions of the ATFL. Thus, this study established 9 subject-specific FE models to explain the injury biomechanism under acute injury, chronic injury, and intact conditions of the ATFL.

Recently, ARFI imaging has been applied in detecting and characterizing a wide variety of soft tissue lesions *in vivo*, including liver fibrosis (Bota et al., 2012), breast lesions (Meng et al., 2011), and thyroid nodules (Zhan et al., 2015). Furthermore, the detection of apparent elasticity changes in the tissues due to different mechanical environments can be measured clinically with sufficient sensitivity using ARFI imaging (Schrier et al., 2020). The principle of ARFI imaging was to use short-duration acoustic radiation forces to generate localized displacements in tissue, typically on the order of 10 μm, and ultrasonic correlation-based methods tracked the localized displacements, which were inversely proportional to the local tissue stiffness (Nightingale et al., 2002). Thus, stiffer tissues led to less local displacement. The apparent elastic modulus (28.04 kPa) of normal ATFL using ARFI imaging calculated with the conventional equation ( $E=3\rho v^{2}$ , ρ is density, v is SWV and E is apparent elastic modulus) represented the linear estimation in micrometer scale deformation for hyperelastic ATFL.

While the elastic modulus of normal ATFL (255 MPa) which was significantly larger than that measured using ARFI imaging applied in the FE model for ADT simulation was assumed with the material properties using the biomechanical test *in vitro* under large deformation in millimeter scale (Funk et al., 2000). Previous literature also noticed the significant discrepancy between the elastic modulus measured by elastography and that by *in vitro* mechanical test. The elastic modulus of the nerve in the toe region measured by the indentation test was significantly smaller than that measured by the elastography (Schrier et al., 2020). However, in the foot FE model developed by Mo et al., the heel fat pad elasticity obtained from elastography showed high consistency with that obtained through the indentation test (Mo et al., 2019). The ratio of the apparent elastic modulus obtained from elastography to that measured from the conventional mechanical test varies in different tissues and perhaps increases with the increase of soft tissue stiffness. Previous literature about elastography assessment of ATFL have reported SWV values ranging between 1.79 m/s (Hotfiel et al., 2018) and 7.6 m/s (Chen et al., 2021) for the healthy controls. The mean SWV value of ATFL in the control group in this study was within the variation range of the literature data mentioned before.

The ADT process was simulated through the FE models with the subject-specific three-dimensional structures and the elastic modulus of ATFL in this study. The simulated AAJS was slightly larger in the acute group, whereas it was significantly smaller in the chronic group, compared to the control group. Additionally, the AAJS decreased significantly with the decrease of the ATFL thickness instead of the elastic modulus indicating that AAJS was mainly affected by ATFL thickness rather than the elastic modulus. The mean thickness of the intact ATFL was reported to be 2.19 mm (Dimmick et al., 2008), similar to our measurement in this study. A previous study that examined 39 CAI patients through MRI and arthroscopy found that 75% of participants’ ATFL were thin (less than 1 mm) or absent (Kanamoto et al., 2014). The chronic patients in our study showed a similar ATFL thickness of 1.08 mm. Although the AAJS slightly increased in the acute group, it was not easily perceived due to the acute pain or instant conservative treatment with immobilization. The AAJS significantly decreased over 3 months after ATFL injury. It has been reported that individuals with a history of ankle sprain have an approximately 3.5 times greater risk of sustaining recurrent ankle sprain compared to those who have no such history (Kucera et al., 2016), indicating an elevated risk of CAI. An observational study about re-sprains during the 12 months of follow-up after an initial ankle sprain suggested that up to 55% of individuals sustained instability, and 28% of the participants reported at least one re-sprain (van Middelkoop et al., 2012). Previous studies have observed ATFL thickness differences in ligament injury, which can be detected using ultrasound in B-mode and MRI but without clear diagnosis criteria of ligament thickness (Kanamoto et al., 2014; Chen et al., 2021). The diagnostic criterion for the acute posterior cruciate ligament injury was larger than 6.5 mm (Wang et al., 2017). Few studies determined the diagnostic criterion in ATFL injury. When the ATFL thickness was less than approximately 1.5 mm when ATFL elastic modulus was under the condition of physiological and pathological conditions, the AAJS start to decrease significantly with over 10 percent of the maximum AAJS, as predicted by the empirical equation fitted in this study. Thus, the ATFL thickness might be a potential biomarker for CAI diagnosis in the clinic.

Despite providing further insight into the relationship among AAJS, thickness, and elastic modulus of ATFL, the subject-specific FE study has the following limitations. The ankle ligaments, except for ATFL, were modeled as numerous wires with 1D truss property, which may compromise simulation accuracy. Due to applying the specific elastic modulus of ATFL in the FE model, which was measured by ARFI non-invasively, the hyperelastic and viscoelastic properties of the ATFL were neglected (Provenzano et al., 2001). The constitutive equation that we proposed was estimated based on FE simulations which ignored the interaction term between thickness and the elastic modulus and other covariates. Additionally, the outcome of the FE models was the talus displacement, which did not take into consideration of the cartilage contact (Alonso-Rasgado et al., 2017). Furthermore, the results in this study are from geometrically specimen-specific FE models, which may hinder external validity or the generalizability of the findings (Wong et al., 2021). Future work will expand to establish a population-based FE model with a 3D structure of cartilage and other ankle ligaments and take into consideration of the heterogenetic properties of bone and hyperelastic and viscoelastic properties of the ATFL.

## Conclusion

The equation proposed in this study described the quantitative dependence of AAJS on the ATFL thickness and elastic modulus. And the thickness of the ATFL may be the dominant factor influencing AAJS, which might be the biomarker for CAI diagnosis.

## Data Availability

The original contributions presented in the study are included in the article/Supplementary Material, further inquiries can be directed to the corresponding author.
